# Aldosterone in Gynecology and Its Involvement on the Risk of Hypertension in Pregnancy

**DOI:** 10.3389/fendo.2019.00575

**Published:** 2019-08-23

**Authors:** Chiara Sabbadin, Alessandra Andrisani, Guido Ambrosini, Luciana Bordin, Gabriella Donà, Jacopo Manso, Filippo Ceccato, Carla Scaroni, Decio Armanini

**Affiliations:** ^1^Department of Medicine–Endocrinology, University of Padova, Padua, Italy; ^2^Department of Women's and Children's Health, University of Padova, Padua, Italy; ^3^Department of Molecular Medicine-Biological Chemistry, University of Padova, Padua, Italy

**Keywords:** aldosterone, polycystyc ovary syndrome, hypertension, preeclampsia, spironolactone, endometriosis

## Abstract

Aldosterone is the main mineralocorticoid hormone, responsible of the regulation of fluid and electrolyte balance and blood pressure. It acts also as a pro-inflammatory factor responsible of an increased cardiovascular risk, independent from blood pressure values. After the discovery of mineralocorticoid receptor (MR) in mononuclear leukocytes, further studies supported its role in inflammatory and even autoimmune mechanisms underlying several diseases. In particular, recent studies reported a possible involvement of aldosterone in some gynecological conditions and diseases, characterized by inflammation, hypertension and increased cardio-metabolic risk, such as use of hormonal contraceptives, preeclampsia, polycystic ovary syndrome, uterine fibroids, and endometriosis. The aim of this mini-review is to report the possible involvement of aldosterone in all these gynecological conditions, suggesting different pathogenetic mechanisms and new target treatments of MR blockers for these diseases.

Aldosterone (Aldo) is the main mineralocorticoid hormone, synthesized and secreted by the zona glomerulosa of the adrenal cortex in response to angiotensin II and potassium. It was discovered in 1953, few years later the discovery of deoxycorticosterone (DOC), the first mineralocorticoid synthesized and studied for its role on electrolyte balance (1). In 1949 Selye, describing the adaption syndrome, hypothesized that the effect of mineralocorticoids was opposite to that of glucocorticoids being cortisol an anti-inflammatory and DOC a pro-inflammatory hormone (2). Subsequently the putative inflammatory role of Aldo was forgotten and all the studies were focused on its role in sodium balance and hypertension. Only in the last decades several studies reported its involvement in cardiovascular and metabolic disorders, revaluating the initial hypothesis of Selye on Aldo as a pro-inflammatory hormone.

Inflammation is a complex systemic process involving blood vessels, immune cells and molecular mediators. It is involved not only in response to harmful triggers, such as pathogens, damaged cells or toxic agents, but also in autoimmunity, somatic mutations, and cancer (3). In 1985 we characterized mineralocorticoid receptor (MR) in mononuclear leukocytes (MNL) and demonstrated that Aldo can regulate the intracellular concentration of electrolytes and the volume of MNL (4–7). In 2004 we reported that incubation of MNL with Aldo can induce *in vitro* the protein expression of some inflammatory markers, as PAI-1 and p22phox (8). This effect was reversed by coincubation with canrenone, the main active metabolite of spironolactone (SP), a MR blocker. Aldo can exert its effects in non-epithelial cells, such as cardiomyocytes, endothelial cells, vascular smooth muscle cells (VSMC) and mesangial cells, leading to fibrosis and remodeling in the heart, vasculature, and kidney. In particular, at the level of the vessel Aldo cross talks with angiotensin II and endothelin-1, inducing inflammation and oxidative stress, regulating cell proliferation, and leading to endothelial dysfunction (9). These effects are mediated through genomic and non-genomic pathways in a MR-dependent or independent manner, as demonstrated for example on erythrocytes, where Aldo induced in a dose- and time-dependent manner membrane alterations, almost completely prevented by canrenone or cortisol (10, 11). These membrane alterations were observed even in erythrocytes of patients affected by primary aldosteronism (PA) and led to increased autologous IgG binding, responsible for premature erythrocyte removal from circulation.

Further studies demonstrated that PA patients present an increased risk of stroke, myocardial infarction, atrial fibrillation, and even metabolic syndrome compared with appropriately matched controls with essential hypertension (12, 13). In fact, Aldo is also involved in the development of several metabolic alterations, impairing insulin secretion and sensitivity, altering potassium levels, increasing inflammatory cytokines and reducing adiponectin (14). Treatment with MR blockers improves cardiovascular and metabolic outcomes similar to surgical treatment of PA (14, 15). In the end of 1990th Pitt and coll. confirmed the importance of MR blockers to prevent cardiovascular complications and cerebrovascular accidents even in patients with normal values of Aldo (16, 17) and several studies are evaluating a possible terapeuthic role of MR blockers to treat obesity and metabolic disorders (18).

Recently, Herrada et al. reported that T-cells are also regulated by Aldo, promoting CD4 T-cell activation and T-helper 17 cell formation (19). T-helper 17 are a subset of CD4 T-cells producing interleukin 17, that is involved in many autoimmune diseases, such as Hashimoto's thyroiditis (20). Recently, we reported that PA patients have an increased prevalence of Hashimoto's thyroiditis (21) and an increased titer of autoantibodies against the angiotensin II type 1 receptor (AT1-AA), that could have some pathogenetic role in PA and related complications (22). From all these studies the important role of Aldo in the genesis of inflammation and related diseases seems mostly mediated by the presence of MR in MNL and macrophages.

The aim of this mini-review is to report the involvement of Aldo in the hypertension induced by pregnancy and in other gynecological diseases frequently associated with hypertension and inflammatory dysregulation.

## Aldosterone in Pregnancy and Preeclampsia

During physiological pregnancy, Aldo levels are increased to induce plasma volume expansion, crucial for maintaining circulating blood volume, blood pressure, and uteroplacental perfusion. These mechanisms are supported already from the first trimester by increased renin production by kidney in response to several cardiovascular adaptations and by increased angiotensinogen secretion by the liver driven by placental production of estrogens (23). This activation of the renin angiotensin Aldo system (RAAS) during pregnancy increases 3- to 7-fold compared to initial values. Further studies have also demonstrated an increased Aldo-to-renin ratio (ARR) in physiological pregnancy, suggesting additional factors which stimulate Aldo production and one of them could be the vascular endothelial growth factor (VEGF) (24). Aldo levels remain high throughout pregnancy, suggesting a possible role in the regulation of placental and fetal development (25). Despite increased Aldo levels, healthy pregnant women do not usually present hypertension through several compensatory mechanisms. The MR-antagonist actions of progesterone and the increased glomerular filtration rate induce natriuresis despite the sodium retaining properties of Aldo. Moreover, in physiological pregnancy angiotensin II shows a reduced pressor effect.

In gestational hypertension and in preeclampsia (PE), differently from physiological pregnancy, decreased RAAS activity and increased sensitivity to angiotensin II effects are found, resulting in decreased plasma volume expansion, vascular constriction, reduction of cardiac output and decreased renal and placental blood flow (23). In particular, PE is the most severe form of hypertension in pregnancy. It is characterized by the new onset of hypertension and proteinuria usually after 20 weeks of pregnancy. The pathogenesis is still debated and remains unknown. Genetic, epigenetic, inflammatory, and autoimmune causes have been involved in the pathogenesis. Several predisposing factors have also been reported, such as chronic arterial hypertension, chronic kidney disease, obesity, diabetes, polycystic ovary syndrome (PCOS), endometriosis, familial or personal history of PE (26). Besides placental abnormalities, many studies suggest a possible role of the RAAS in the pathogenesis of PE. As previously reported, both Aldo and progesterone are high during pregnancy, but the number of MR in MNL has been found normal in physiological pregnancy and low in PE (27), as reported in patients with PA (28). More interestingly the effector mechanism of Aldo, measured through the potential difference between rectal and oral mucosa, was normal in normal pregnancy and high in PE, as previously reported in PA (29). We hypothesized that some factor produced by the placenta blunts the effect of increased Aldo in normal pregnancy, but it lacks in PE, leading to the classical effects of Aldo excess, even though Aldo levels are reduced. Other possible explanations could be a reduced antagonist effect of progesterone at the level of MR in PE (30) or an impaired VEGF-mediated stimulation of aldosterone synthase (24). From these considerations a possible therapeutic role of MR blockers in PE has been hypothesized (31). Unfortunately, SP has a potent antiandrogen effect and both canrenone and potassium canrenoate have a weaker antiandrogen action, which could alter male fetus morphogenesis (32, 33). Eplerenone could be the preferred MR blocker in pregnancy, showing no-antiandrogen effect (31). Indeed, isolated reports show its efficacy to manage blood pressure in PA during pregnancy (34). However, women with PA usually show an improvement in blood pressure values and hypokaliemia during pregnancy, probably because of the antagonist effect of increased progesterone levels at the level of MR (35).

Another interesting hypothesis of the pathogenesis of PE was postulated by Wallukat et al. (36), who firstly described in preeclamptic women the presence of AT1-AA, which were able to induce a preeclamptic state when injected in pregnant mice (37). The subsequent discovery of increased titers of AT1-AA even in PA patients (22) leads to a possible common underlying etiopathogenesis of PE and PA. The possible pathogenetic role of AT1-AA is complex and could be related to the activation of AT1 receptor, leading to increased vascular resistance and endothelial damage, due to increased production of many pro-oxidative and pro-inflammatory factors. The interplay between placental abnormalities, oxidative stress and endothelial dysfunction are key events in the development of PE, but more studies are needed to better understand the etiology of this complex disease (38).

## Aldosterone in other Gynecological Conditions

Recent studies have reported a possible involvement of Aldo in other gynecological diseases, sometimes associated with inflammation, hypertension and actual or future cardio-metabolic risk, such as the use of hormonal contraceptives, PCOS, uterine fibroids and endometriosis.

## Aldosterone and Hormonal Contraceptives

Hypertension was a frequent complication of the hormonal contraceptives with high ethinilestradiol content and it is actually a rare side effect of contraceptives with lower amounts of ethinilestradiol (20–30 μg), but still reported in some cases, stressing out the implication of a genetic predisposition. It is well known that estrogens increase both cortisol and Aldo. Cortisol increases in response to the increased level of cortisol binding globulins in order to maintain its free quote in the normal range. The mechanism involved in the increase of Aldo during contraceptives is not completely clarified. The most likely hypothesis is that estrogens increase the synthesis of angiotensinogen, activating all the RAAS and inducing the onset of sodium and water retention, metabolic alterations and sometimes hypertension (39). For all these reasons, it is recommended to avoid the use of hormonal contraceptives in patients with hypertension and metabolic alterations.

## Aldosterone and PCOS

PCOS is a very common disease affecting about 10% of women in the reproductive age. It is characterized by oligo-anovulation and biochemical/clinical signs of hyperandrogenism. Several phenotypes have been described, but a common feature is insulin resistance, interesting about 70% of patients even normoweight and representing one of the main pathogenetic factors of the syndrome (40). Beyond the reproductive disorders, even at an early stage PCOS patients can present a clustering of cardiovascular risk factors, such as insulin resistance, obesity, diabetes, dyslipidemia, hypertension, endothelial dysfunction, and low-grade chronic inflammation (41). Previous studies found that Aldo and ARR, though normal and not consistent with PA, are higher in PCOS women compared with age- and body mass index-matched healthy controls and correlate with blood pressure values and some metabolic and cardiovascular markers (42, 43). As previously reported, Aldo plays a key role as pro-inflammatory hormone and cardiovascular and metabolic risk factor (12, 13) and could play a certain role in the development of many Aldo-related disorders even in PCOS, such as hypertension, PE, metabolic alterations, Hashimoto's thyroiditis and cardiovascular diseases. Moreover, we recently reported that PCOS erythrocyte membranes showed an increased oxidized level and enhanced sensitivity to oxidative injuries (41, 44), as reported in PA patients' erythrocytes, confirming a systemic inflammatory status (10, 11).

The treatment of PCOS should take into consideration not only the acute manifestations of the syndrome, like menstrual disorders and hirsutism, but also the related long-term cardiac and metabolic complications, that could be worsened in pregnancy and after menopause. Hormonal contraceptives are first-line management for the menstrual abnormalities and hyperandrogenic disorders of PCOS patients (45). However, their role on insulin resistance is controversial and considering their activation of RAAS, it could be hypothesized a further increase of Aldo levels in PCOS patients, that could worsen some cardio-metabolic aspects of the disease. Therefore, their use should be carefully evaluated for every single patients, after considering all the pre-existing metabolic and cardiovascular risk factors. We have reported that SP is useful both in controlling the clinical signs of hyperandrogenism and in preventing the future cardiovascular risk (46). Its main side effects are hypotension and hyperkalemia, due to MR block, and frequent menstrual abnormalities, due to a complex effect on estradiol and progesterone actions (47). Because of the potential risk to the male fetus due to its antiandrogen effect, women must be informed to take precautions to avoid pregnancy during SP treatment. The association of SP with hormonal contraceptives is useful for many reasons: it enhances the antiandrogen effects, it prevents unwanted pregnancies and menstrual abnormalities linked to SP and it reduces the side effects related to the activation of RAAS induced by some contraceptives, such as sodium and water retention, but even hypertension and cardio-metabolic alterations. When hormonal contraceptives are controindicated, the addition of low dose progestins from the 14th day of the cycle can block the bleeding induced by SP (46, 47) and the use of licorice can prevent hypotension and hyperkalemia, blocking the 11β-hydroxysteroid dehydrogenase type 2, and can enhance the antiandrogen effect, blocking the 17-hydroxysteroid dehydrogenase and 17–20 lyase in adrenals and ovaries (48–51).

## Aldosterone and Uterine Fibroids

Uterine fibroids (UF) are benign tumors of the muscle layer of uterus, affecting about 70% of women by age 50 years but being symptomatic only in half of the cases. UF are frequently associated with familial predisposition, obesity, early menarche, low parity, and are influenced by the effect of several hormones. Progesterone stimulates cellular proliferation and UF growth (52) and the treatment with progesterone receptor (PR) antagonist mifepristone (RU486) or with the selective PR modulator ulipristal acetate is effective in reducing UF size and collagen deposits and in controlling uterine bleeding (53). Local and general inflammation have been associated with UF development and T-helper 2 bias in the uterine environment has been hypothesized to contribute to UF formation, independent of the sex steroid status (54). A recent study has evidenced an involvement of Aldo in UF, showing a significant stimulatory effect on leiomyoma cell proliferation, which was blocked by the pre-incubation with SP or eplerenone (55). These *in vitro* studies suggest a possible use of MR blockers in the treatment of UF; however, only eplerenone could be considered, because of the frequent metrorrhagia associated with SP (47). Recently a higher risk of hypertension has been reported in patients with UF (56). However, a previous study evidenced that hysterectomy did not influence the risk of hypertension and on the contrary it was associated with long-term increase of cardiovascular risk (57). These studies are consistent with possible extra-uterine mechanisms involved in hypertension in UF. It is unlikely an involvement of progesterone because of its MR antagonism, while Aldo could be the pathogenetic factor common to hypertension and UF and could explain the association of UF with other Aldo-related disorders, such as insulin resistance, obesity and PCOS (58).

## Aldosterone and Endometriosis

Endometriosis is a complex syndrome characterized by an estrogen-dependent chronic inflammatory process, caused by the presence of endometrial-like tissue out the uterine cavity. It is the most common cause of chronic pelvic pain in women and it is associated with infertility. Several studies have underlined an important role of inflammation in the pathogenesis of the disease (59). Chronic inflammation is a risk factor for several diseases, as atherosclerosis, diabetes, hypertension, hypothyroidism, autoimmune diseases and cancer and it is reported that endometriosis is more frequently associated with all these disorders (60). Hormonal abnormalities or chronic systemic inflammation characterizing endometriosis may result in a higher risk of hypercholesterolemia and hypertension. Conversely, hypercholesterolemia and chronic systemic inflammation resulting from hypertension may increase the risk of endometriosis (61). A recent study using metabolomics has shown that Aldo is higher in infertile patients with endometriosis (62). As previously demonstrated (8), Aldo could enhance the systemic and local inflammation underlying endometriosis, through the activation of MR present in peritoneal and tissue inflammatory cells, in particular macrophages and MNL. Moreover, Aldo could increase oxidative stress through MR-dependent and -independent pathways in VMSC, that promote proliferation of fibroblasts in the perivascular space (63). A recent study in an animal model of continuous ambulatory peritoneal dialysis demonstrated that SP was effective at decreasing intraperitoneal fibrosis and inflammation (64). Considering the substantial experience with the safe chronic use of SP in women with androgen excess disorders as PCOS, investigators should evaluate the therapeutic potential of SP or other MR blockers in endometriosis. In our experience, SP is also very useful in the dysmenorrhea probably for its powerful anti-inflammatory effect.

## Conclusions

In the recent years Aldo has been considered an important pro-inflammatory hormone, involved in many conditions characterized by chronic inflammation, as cardio-metabolic and some gynecological disorders. Inflammation starts in the circulation activating MNL and macrophages, that migrate in inflammatory tissues, presenting their MR that can be activated by Aldo, enhancing the inflammation (5–7, 63). The finding of a reversal of inflammatory properties of Aldo by coincubation or treatment with MR blockers is a further confirmation of the important role of Aldo. In the recent years the normal range of Aldo has been revisited (3, 4), stressing the importance of its ratio with renin, that could represent a sign of enhanced Aldo effector mechanism when it is increased (43). Further studies are needed to evaluate the therapeutic potential of MR blockers even as anti-inflammatory drugs, especially in some gynecological diseases characterized by a pro-inflammatory state and associated with hypertension and an increased cardio-metabolic risk, that could be related to Aldo dysregulation.

## Author Contributions

All authors listed have made a substantial, direct and intellectual contribution to the work, and approved it for publication.

### Conflict of Interest Statement

The authors declare that the research was conducted in the absence of any commercial or financial relationships that could be construed as a potential conflict of interest. The reviewer FF declared a shared affiliation, with no collaboration, with several of the authors, AA, GA, LB, and GD, to the handling editor at the time of the review.
